# Automated GMP production and long-term experience in radiosynthesis of the SV2A tracer [^11^C]UCB-J

**DOI:** 10.1186/s41181-025-00377-0

**Published:** 2026-05-19

**Authors:** Saara Wahlroos, Anna Krzyczmonik, Semi Helin, Johan Rajander, Olof Solin, Anna K. Kirjavainen

**Affiliations:** 1 Radiopharmaceutical Chemistry Laboratory, Turku PET Centre, University of Turku, Turku, Finland; 2 Accelerator Laboratory, Turku PET Centre, Åbo Akademi University, Turku, Finland; 3 Department of Chemistry, University of Turku, Turku, Finland

**Keywords:** [^11^C]UCB-J, GMP, SV2A, Carbon-11, PET radiochemistry, Suzuki–Miyaura cross-coupling, Radiopharmaceutical, Automation, SPE, radioHPLC

## Abstract

**Background:**

Positron emission tomography (PET) is a non-invasive imaging technique that enables the quantification of specific biological and pharmacological processes in vivo. PET can be used to investigate synaptic density by targeting synaptic vesicle glycoprotein 2A (SV2A), and can measure brain levels of SV2A in patients with epilepsy and other neurologic, neurodegenerative, or psychiatric conditions that involve SV2A. The well-known radiotracer [^11^C]UCB-J ((*R*)-1-((3-[^11^C]methylpyridin-4-yl)methyl)-4-(3,4,5-trifluorophenyl)pyrrolidin-2-one) targets SV2A and thereby enables visualization of synaptic density in vivo.

**Results:**

[^11^C]UCB-J synthesis was performed using [^11^C]methyl iodide ([^11^C]CH_3_I) produced with a commercial device. Here, we present an in-house-built device for [^11^C]UCB-J production from [^11^C]CH_3_I and a suitably treated boronated precursor. The whole radiosynthetic procedure was automated, and has now been in clinical production according to GMP for more than four years. We also address several issues encountered during the production process development and tracer utilisation.

**Conclusions:**

The developed method enables robust [^11^C]UCB-J production, with a yield of 2.8 ± 0.6 GBq and molar activity of 150 ± 51 GBq/µmol (n = 56) at end of synthesis. The product fulfils all specifications set for a clinical tracer. GMP regulations and guidelines in all aspects of radiopharmaceutical preparation were followed. Although [^11^C]UCB-J production is challenging, good and reliable radiochemical yields can be obtained by using a carefully designed production protocol.

## Background

Positron emission tomography (PET) is a non-invasive functional and metabolic imaging technique that allows the quantification of specific biological and pharmacological processes in vivo. For example, PET can be used to study the functions of neurotransmitters in the central nervous system (CNS) (Ametamey et al. 2008; Strauss and Conti 1991). Altered synapse structure or synaptic loss can result in dysfunctional neuronal signalling (Finnema et al. 2016). Synaptic pathology is associated with many neurological disorders, including epilepsy (Van Vliet et al. 2009), Alzheimer’s disease (Hamos et al. 1989), autism (Tang et al. 2014), depression (Kang et al. 2012), and schizophrenia (Glantz and Lewis 2000). PET can be used to investigate synaptic density by targeting synaptic vesicle glycoprotein 2A (SV2A) (Finnema et al. 2016), which is a transmembrane protein expressed in secretory vesicles and present in all brain areas (Bajjalieh et al. 1994). Notably, SV2A is the target for the antiepileptic drug levetiracetam (Lynch et al. 2004), and SV2A dysfunction has been implicated in other neurologic disorders. PET imaging can be performed to measure brain levels of SV2A in patients with epilepsy and other neurologic, neurodegenerative, or psychiatric conditions involving SV2A (Finnema et al. 2016; Milicevic Sephton et al. 2020).

The radiotracer [^11^C]UCB-J ((*R*)-1-((3-[^11^C]methylpyridin-4-yl)methyl)-4-(3,4,5-trifluorophenyl)pyrrolidin-2-one) has excellent in vitro and in vivo properties for imaging SV2A, and thereby enables visualization of synaptic density in vivo. This radiotracer can also be used to measure drug binding to its target, and PET imaging of SV2A can be performed to characterize the binding of levetiracetam or other SV2A-targeting drugs (Finnema et al. 2016; Milicevic Sephton et al. 2020; Nabulsi et al. 2016). The synthesis of [^11^C]UCB-J was reported in 2014 and 2016 by Nabulsi et al. The radiosynthesis of [^11^C]UCB-J was originally performed via Suzuki–Miyaura cross-coupling of [^11^C]methyl iodide ([^11^C]CH_3_I) with the 3-pyridyl trifluoroborate precursor containing 3–10% boronic acid (Suzuki 1991; Ishiyama et al. 1993; Miyaura and Suzuki 1995). While this synthesis resulted in good radiochemical yield (RCY), it couldn’t be directly implemented for good manufacturing practice (GMP) production due to the degradation over time of the boronic acid. However, the use of purified precursor resulted in drastically decreased RCY. In 2017, Onega et al. identified in situ produced boronic acid containing precursor as the more effective labelling precursor, and presented a procedure for generating boronic acid from the GMP grade 3-pyridyl trifluoroborate precursor immediately prior to [^11^C]UCB-J synthesis. In 2020, Milicevic Sephton et al. reported several changes to this acid pre-treatment procedure. In 2019, Rokka et al. developed a simplified method for [^11^C]UCB-J synthesis, by using a different solvent for the reaction and thereby making it a one-pot reaction (Rokka et al. 2019, 2021).

**Scheme 1:**
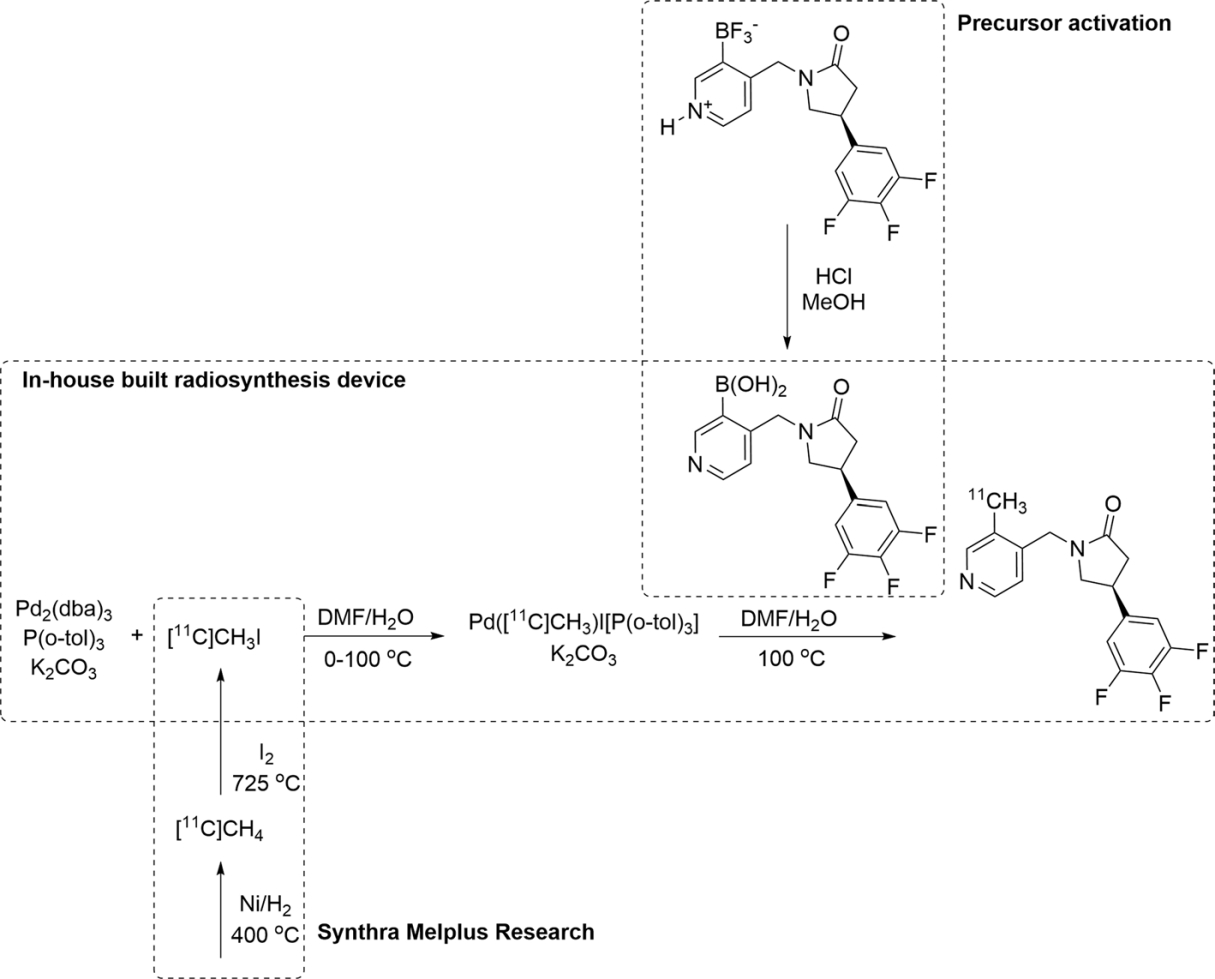
Synthesis of [^11^C]UCB-J. Precursor activation is conducted outside of the clean room prior to synthesis. The Synthra MeI plus Research device and in-house-built radiosynthesis device are located in separate hot cells in the clean room, and are connected with a capillary for [^11^C]CH_3_I delivery

A fully automated device for [^11^C]UCB-J synthesis has been built, and the reproducible and automated radiosynthesis of [^11^C]UCB-J has been realised with high RCY and radiochemical purity (RCP), in compliance with GMP regulations. The radiosynthesis of [^11^C]UCB-J (Scheme 1) is performed via Suzuki–Miyaura cross-coupling of the acid pre-treated 3-pyridyl trifluoroborate precursor with [^11^C]CH_3_I.

Recently several useful guideline articles were published (Todde et al. 2017; Gillings et al. 2020, 2021) giving guidance for preparation and validation as well as validation of analytical methods for small-scale radiopharmaceuticals (aka. small-scale "in-house" radiopharmaceuticals; SSRP). General and particular aspects on preparation of radiopharmaceuticals from non-licensed components are presented and discussed in depth. In our particular case we present the preparation of a SSRP from non-licensed starting materials, involving several complex chemical manipulations and purification steps and sterile filtration in the end. Overall, we have followed the guidance and regulations of EU GMP (Eudralex, Volume 4 Good manufacturing practice (GMP) Guidelines).

## Materials and methods

### Personnel

The staff performing the synthesis and quality control were properly educated and trained according to EU GMP regulations. The roles and responsibilities of the quality assurance (QA) unit and responsible person for radiopharmaceuticals (RPR) were according to EU GMP regulations (Eudralex, Volume 4 Good manufacturing practice (GMP) Guidelines).

### Chemicals and materials

The precursor compound (*R*)-3-(difluoroboranyl)-4-((2-oxo-4-(3,4,5-trifluorophenyl)pyrrolidin-1-l)methyl)-pyridin-1-ium fluoride (GMP grade) and the reference compound (4*R*)-1-[(3-methyl-4-pyridyl)methyl]-4-(3,4,5-trifluorophenyl)pyrrolidin-2-one were supplied by PharmaSynth AS (Tartu, Estonia). Adequate specifications and analytical documentation for these were received and checked. All other chemicals and materials were approved based on their certificates obtained from their respective vendors. N,N-dimethylformamide (DMF; anhydrous, 99.8%), tris(dibenzylideneacetone)dipalladium (Pd_2_(dba)_3_; 97%), tri(o-tolyl)phosphine (P(o-tol)_3_; 97%), and hydrochloric acid (HCl; ACS reagent, 30%) were obtained from Merck (Dramstadt, Germany). Sterile water (aqua sterilisata, i.v.) was obtained from B. Braun Melsungen (Melsungen, Germany), ethanol (European Pharmacopoeia, Ph. Eur.) from Berner (Helsinki, Finland), 1 M hydrochloric acid (HCl; 0.9983 ± 0.0038 mol/l) from FF-chemicals (Haukipudas, Finland), and sodium chloride (NaCl) solution (9 mg/mL, sterile, i.v.) from B. Braun Melsungen (Melsungen, Germany). Ammonium formate (NH_4_COOH; ≥ 97%), acetonitrile (≥ 99.9%), methanol (≥ 99.9%), and potassium carbonate (K_2_CO_3_; ≥ 99%) were obtained from Honeywell (Muskegon, MI, USA).

Solid phase extraction (SPE) cartridges (Sep-Pak C18 Plus Light, WAT023501), were obtained from Waters Corp. (Milford, MA, USA). Sterile Millex-GP filters with polyethersulfone membrane (0.22 µm, 33 mm) used for sterile filtration were obtained from Merck Millipore Ltd (Carrigtwohill, Ireland). Stainless steel (SS) frits (10-micron), used for filtration of the crude synthesis solution prior to preparative separation, were obtained from Analytical Scientific Instruments US (Richmond, CA, USA). Iodine (sealed ampules, 5 g or 25 g, 99.999%) for the iodine reactor was obtained from Sigma-Aldrich (Saint Louis, MO, USA). Nickel (powder, Puratronic 99.999%, low carbon; C < 100 ppm) for the nickel oven was from Thermo Scientific (Thermo Fisher GmbH, Kandel, Germany). The Porapak Q (50–80 mesh) in the [^11^C]CH_3_I trap was from Waters Corp. (Milford, MA, USA).

### Chromatographic methods

Semi-preparative radio-HPLC (high-performance liquid chromatography) was performed using a UFLC Shimadzu Prominence liquid chromatograph pump (LC-20AD; Shimadzu), a pre-column SecurityGuard SemiPrep Cartridge Gemini-NX C18 between the injector and column inlet (5 µm, 10 × 10 mm; Phenomenex, Milford, MA, USA), and a Gemini-NX C18 110 Å column (5 µm, 10 × 250 mm; Phenomenex). A miniature Geiger-Müller (GM) tube, for radioactivity monitoring, was placed at the column outlet. Isocratic elution was done using two different protocols. Protocol 1; NH_4_COOH (0.1 M aq., pH = 10; NH_3_):CH_3_CN (v/v, 65:35) was used with a flow rate of 7.0 mL/min. In protocol 2; initially, the elution was by pure eluent A: NH_4_COOH (0.1 M aq., pH = 10; NH_3_) for two minutes and then changed to eluent B: NH_4_COOH (0.1 M aq., pH = 10; NH_3_):CH_3_CN (v/v, 65:35). The flow rate was 9 mL/min throughout. The retention time of [^11^C]UCB-J was 11.5 ± 1.0 min with protocol 1 and 13.0 ± 0.5 min with protocol 2. During process development a UV detector (Merck Hitachi L-4000, λ = 220 nm) was placed at the column outlet. Analytical radio-HPLC was performed using a Hitachi L-2000 series HPLC pump equipped with a VWR-Hitachi L-2400 UV-adsorption detector (λ = 261 nm), a 2 × 2-inch NaI radioactivity detector in series at the column outlet, and a Gemini C18 column (3 µm, 4.6 × 100 mm; Phenomenex). Isocratic elution with NH_4_COOH (0.1 M aq., pH = 4.2; HCOOH):CH_3_CN (v/v, 62:38) was used with a flow rate of 1.0 mL/min. The retention time of [^11^C]UCB-J was 4.3 ± 0.4 min.

### Synthesis devices

**Fig. 1:**
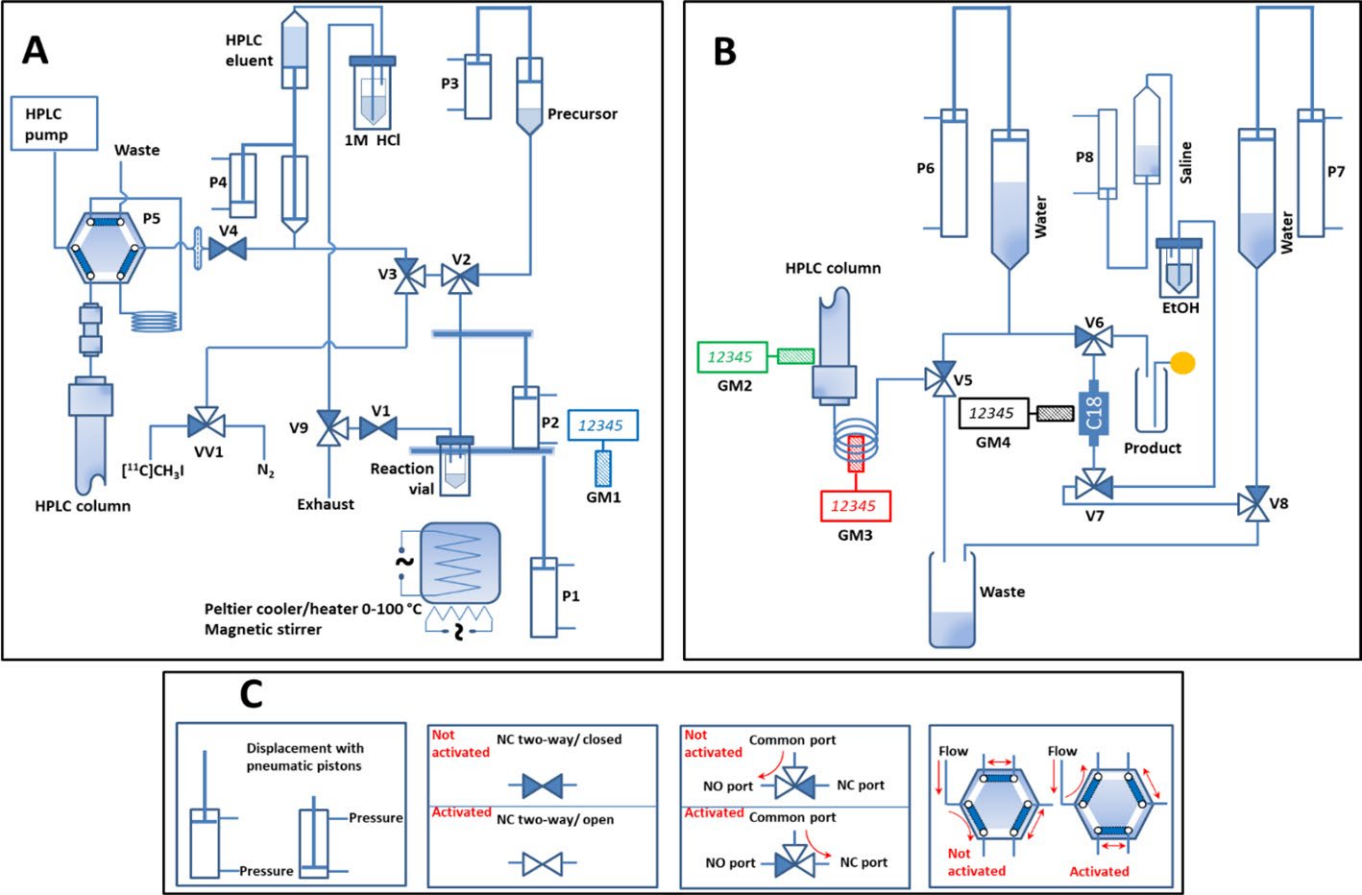
Schematic diagram of the synthesis device for the production of [^11^C]UCB-J from [^11^C]CH_3_I. **A** The reaction vessel and Peltier cooler/heater are depicted in the lower right corner of the panel. Vessels for precursor and dilution solutions are shown as well as injection system for HPLC purification. **B** Collection of the HPLC fraction and solvent exchange unit with solid-phase extraction cartridge. **C** Explanations of the valves and pistons are presented (NO; normally open, NC; normally closed)

The automated synthesis involved [^11^C]CH_3_I production using the Synthra MeIplus Research device (Synthra GmbH, Hamburg, Germany) and an in-house built device for the [^11^C]UCB-J labelling reaction and purification (Fig. 1). Both devices were subject to installation qualification (IQ) and operational qualification (OQ) for their intended purposes. The in-house built synthesis device utilized pneumatically operated stainless steel or electrically operated PEEK two- and three-port valves, and a pneumatically operated SS six-port distribution valve for liquid and gas path control. Liquid transfer was achieved using single-use plastic syringes connected to pneumatically operated pistons. The reaction vial was cooled and heated by a Peltier element with a temperature range of 0–100 °C (heated from 0 °C to 100 °C in approximately 3 min). This temperature range enabled the efficient trapping of [^11^C]CH_3_I at 0 °C, complexation (30–100 °C), and labelling reaction (100 °C). Ethylene glycol was used for good thermal conductivity between the device walls and reaction vessel. Beneath the Peltier device, a small magnetic stirring plate was installed, to enable stirring of the reaction mixture at all times.

The system included an HPLC injector and a semi-preparative HPLC column. A sterile filtration unit (SFU) was used for sterile filtration and the sterile filter integrity test (pressure hold test, PHT). An in-house-built control system was installed on a computer outside the hot cell, and this was used to operate the synthesis device as described before by Savisto et al. (2017). The HMI/SCADA software used to operate the synthesis device was iFix (GE Vernova Inc., Cambridge, Massachusetts, USA). An automatic synthesis sequence was programmed. During the synthesis sequence, the human operator can intervene with the commands “pause”, “continue”, and “skip”. The “pause” command halts the synthesis sequence at the certain step and the “continue” command continues the sequence from the paused step. The “skip” command forces a jump to the following step.

The synthesis device, HPLC injector, semi-preparative column, and Peltier element were placed inside the same hot cell situated in a clean room (EU grade C). The hot cells and premises were validated and qualified as well as operated according to the EU GMP regulations (Eudralex, Volume 4 Good manufacturing practice (GMP) Guidelines). The Synthra MeIplus Research device and the SFU were placed in separate hot cells. The [^11^C]CH_3_I produced in the Synthra device was transferred to the synthesis device through a teflon capillary (OD 1/16 inch, ID 1 mm, length ~ 8 m) connecting these two devices.

### Pre-synthesis preparations

Before synthesis, as detailed below, the gas transfer lines of the Synthra device were flushed with helium and hydrogen, in order to remove any carbon impurities and to condition the nickel catalyst, thus ensuring high molar activity (A_m_). The flushing sequence comprises flushing of the circulation part containing [^11^C]CH_4_ and [^11^C]CH_3_I traps with helium, and flushing of the [^11^C]CO_2_ trap and the nickel oven with hydrogen. The circulation part was pressurized with helium, the circulation pump was started, and the pressure was released by a vacuum pump. This pressurizing was repeated three times in the flushing sequence. The nickel oven was flushed first without heating, and then while heating to 425 ºC.

Activation of the precursor was conducted prior to the synthesis. The activation was achieved by adding methanol (55 µL) and HCl (22 µL, 30%) to the precursor (1.7 mg), and stirring the mixture for 30 min at room temperature. Next, this mixture was dried in a stream of helium and vacuum, after which the precursor was dissolved in DMF (300 µL) and sterile H_2_O (v/v, 8:1). The precursor solution was taken up into a 1-mL glass syringe, and the syringe was put into place on the device.

The reaction solution was also prepared prior to the synthesis. Pd_2_(dba)_3_ (0.5 mg) was weighed directly to the reaction vessel initially containing a small stirring bar (Cylindrical 8 × 3 mm, VWR, UK) which was later replaced by a micro-magnetic stirrer (Spinbar® magnetic stirring fleas, 2-mm diameter, 2-mm length, SP Bel-Art, Wayne, NJ, USA), and dissolved with DMF (245 µL). P(o-tol)_3_ stock solution (200 µL, 9.5 mg in 4 mL DMF) and K_2_CO_3_ stock solution (55 µL, 17.5 mg in 2 mL sterile H_2_O) were added, and the reaction solution was mixed for 1 min. Then the reaction solution was immediately degassed with helium for 5–10 min. The reaction solution was retained at 0 °C and again mixed for 1 min right before the synthesis.

### [^11^C]Carbon dioxide production

[^11^C]Carbon dioxide ([^11^C]CO_2_) was produced in-target via the ^14^N(p,α)^11^C reaction in nitrogen mixed with up to 0.2% oxygen, with 17.5 MeV protons using an ACSI TR19 cyclotron (Advanced Cyclotron Systems, Inc., Richmond, BC, Canada). The target gas was irradiated for 20 min with a beam current of 40 μA. The produced [^11^C]CO_2_ in nitrogen was delivered to the synthesis device via a 1/16-inch OD, 1.1-mm ID stainless steel (SS) capillary tube using ~ 250 mL/min flow. The transport distance was approximately 60 m.

### [^11^C]CH_3_I production

[^11^C]CH_3_I was produced by the Synthra MeI 3D18061 gas-phase halogenation sequence, with some modifications, as follows here. Iodine temperature was set at 90 °C instead of 95 °C and the CH_4_ trap was at − 140 °C instead of − 120 °C when trapping. Subsequently, the CH_4_ trap was heated to 160 °C (i.e. not to 120 °C) when releasing [^11^C]CH_4_. During desorption of [^11^C]CH_3_I, the helium flow was initially 30 mL/min and was then decreased to 10 mL/min.”

### [^11^C]UCB-J production

[^11^C]CH_3_I from the Synthra platform was transferred through a capillary tube, and bubbled through the reaction mixture cooled to 0 °C. When the transfer was completed, the reaction vessel was heated to 100 °C during the reaction, which was carried out for 3 min. Subsequently, the precursor solution was added, and the reaction was continued for 5 min at 100 °C. Using a double-syringe arrangement (Fig. 1), the reaction solution was first diluted with HCl (1.6 mL, 1 M), and then further diluted with HPLC eluent (3 mL; eluent A in protocol 1 and eluent B in protocol 2) for injection on the semipreparative HPLC-column. Prior to injection on the column, the solution was passed through the SS frit.

**Fig. 2:**
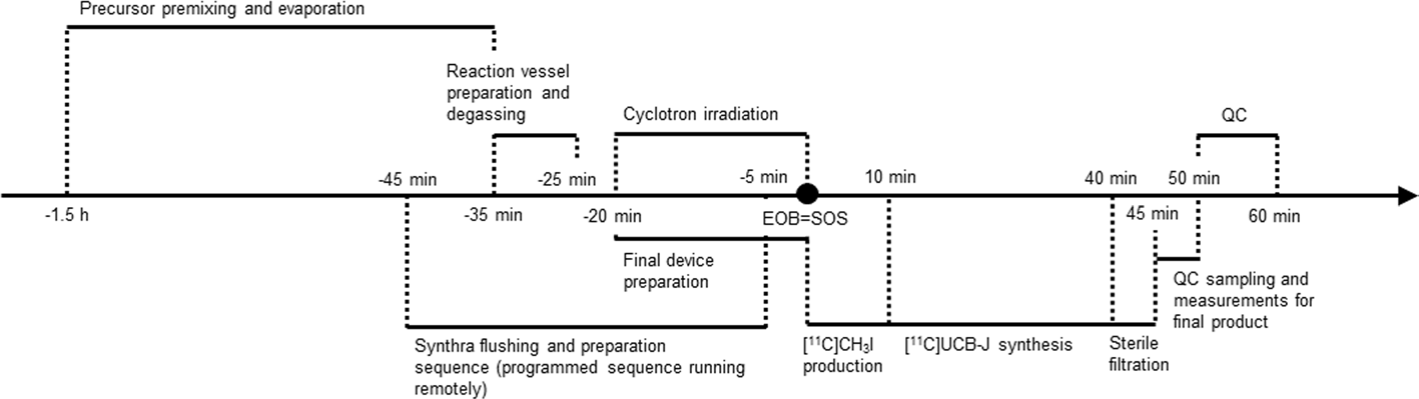
Synthesis timeline. EOB—end of bombardment, SOS—start of synthesis

In protocol 1 the [^11^C]UCB-J was collected from the semi-preparative HPLC column outlet to a syringe containing sterile water (20–25 mL). Based on the radioactivity signal, the collection continued for no longer than 1 min 45 s (14 mL). [^11^C]UCB-J was trapped in an SPE cartridge by pushing the collected HPLC-fraction through the cartridge, and the cartridge was then washed with 15 mL sterile water. [^11^C]UCB-J was eluted from the cartridge into a collection vial with ethanol (0.8 mL), followed by saline (11 mL) for dilution. In protocol 2 this procedure was slightly modified. The [^11^C]UCB-J was collected from the semi-preparative HPLC column outlet to a syringe containing 35 mL of sterile water. The collection of the product fraction was done by the operator, based on the radioactivity signal. Subsequent procedures were as in protocol 1. The product solution was filtered through a sterile non-pyrogenic single-use syringe filter into a sterile end-product vial. Figure 2 presents the synthesis timeline.

### Specifications and quality control (QC) tests

Quality control (QC) tests of the QC sample were performed following EU GMP guidelines (Eudralex, Volume 4 Good manufacturing practice (GMP) Guidelines). The applied HPLC method is described in Section Chromatographic methods. Table 1 presents the specifications and analysis methods for [^11^C]UCB-J.

**Table 1:** Specifications and quality control (QC) test methods

Test	Acceptance criteria	Method	A	B	C
Appearance	Clear, colourless solution, free of particles	Visual inspection	X	X	X
Radiochemical identity	R_t_([^11^C]UCB-J) = R_t_(UCB-J) ± 0.3 min	Liquid chromatography (LC)	X	X	X
Radioactivity	The injection contains ≥ 90.0% and ≤ 110.0% of the declared carbon-11 radioactivity at the date and time stated on the delivery sheet	Measured in dose calibrator	X	X	X
Radiochemical purity (RCP)	The carbon-11 radioactivity in the form of [^11^C]UCB-J is ≥ 95.0%	LC	X	X	X
Radionuclide identity	Half-life of 19.9–20.9 min	Measured in dose calibrator	X	X	X
Chemical purity (CP)	Sum of the content of UCB-J and total chemical impurities is ≤ 10 μg/dose	LC	X	X	X
PH	4.5–6.5	pH indicator strip	X	X	X
Sterile filter integrity	Relative pressure decrease (RPD) < 10%	Pressure hold test	X	X	X
Residual solvents	The concentration of DMF is ≤ 880 ppm, acetonitrile ≤ 410 ppm, and methanol ≤ 3000 ppm	Gas chromatography (GC)	X	X	X
Ethanol content	The end product contains ≤ 10%	GC	X	X	X
Bacterial endotoxins	Endotoxin limit is < 17.5 IU/mL	European Pharmacopoeia (Ph. Eur)	X	X	
Sterility	Sterile according to Ph. Eur	Ph. Eur	X	X	
Palladium content*	The end product contains ≤ 1 μg/mL	Inductively coupled plasma-mass spectrometry (ICP-MS), Ph. Eur	X		
Shelf-Life	To be used within 90 min after synthesis	QC analysis of CP, RCP, pH, and appearance	X		

^A^Performed on the process validation batches

^B^5% of the production runs are tested

^C^Performed on each batch

^*^Performed quarterly

#### Radiochemical yield and molar activity calculations

The reported RCYs were calculated from the amount of [^11^C]CH_3_I trapped in the initial reaction solution and the activity of the final product, with both values decay corrected to the same time-point (Coenen et al. 2017; Herth et al. 2020). Molar activity of the [^11^C]UCB-J final product was determined from the [^11^C]UCB-J concentration (determined by analytical HPLC UV detection) and radioactivity, which was measured from a known sample volume in a dose calibrator and decay corrected to the end of synthesis (EOS) (Coenen et al. 2017).

#### Data analysis and statistics

The results are expressed as the mean group values with standard deviation (SD). The assumption of normal distribution was checked based on the studentised residuals. Differences between the reactions with a big activity loss and normal reactions were tested using the two-tailed unpaired t test. Statistical analyses were performed in GraphPad Prism (GraphPad Software, v. 6.0, San Diego, CA, USA). Differences were considered significant when p < 0.05.

## Results

Our in-house-built device for [^11^C]UCB-J production enabled effective trapping of [^11^C]CH_3_I in the initial reaction mixture, 59 ± 5% (n = 150) of the amount produced with the Synthra device.

Synthesis using Onega’s pre-treatment protocol (Onega et al. 2017) was chosen as starting conditions for the initial experiments. The synthesis protocol was applied without any changes, and the achieved RCY was 11.4 ± 5.0% (Table 2, entry 1). Based on these results, the protocol was further optimised. First, the process of dilution and transfer to the HPLC injection loop was improved. Experiments performed with this transfer system resulted in an increased RCY of 15.5 ± 5.5% (Table 2, entry 2). Second, the shape and size of the reaction vessel was changed. This change resulted in an increased RCY of 23.6 ± 3.9% (Table 2, entry 3). Third, the initial handling of the precursor was addressed. Using 30% HCl for precursor reaction resulted in an increased RCY of 37.9 ± 7.5% (Table 2, entry 4). After the sterile filtration with the SFU, the radioactivity remaining in the filter was less than 10% of the total.

**Table 2:** Results of the optimization experiments. Data are presented as mean ± standard deviation. n = number of trials and RCY = radiochemical yield

Entry	n	RCY (%)	
1	3	11.4 ± 5.0	Starting conditions – Onega’s protocol (Onega et al. 2017)
2	4	15.5 ± 5.5	Improved transfer from reaction vessel to HPLC injector loop
3	7	23.6 ± 3.9	New reaction vessel and increased reaction volume
4	5	37.9 ± 7.5	HCl (30%) used for precursor reaction

Table 3 shows results received during and after process validation. 150 successful syntheses were performed with RCY of 23.3 ± 6.4%. 94 of these syntheses were performed using protocol 1 as preparative HPLC method (RCY 21.1 ± 6.1%) and 56 using new preparative HPLC method protocol 2 (RCY 26.7 ± 5.6%).

**Table 3:** Radiochemical yields and purities, molar activities, and radioactivity amounts achieved at EOS (end of synthesis)

Entry	n	RCY (%)	RCP (%)	A_m_ at EOS (GBq/µmol)	Radioactivity at EOS (GBq)
Results of successful syntheses performed during and after process validation	150	23.3 ± 6.4	99.3 ± 0.2	143 ± 71	2.5 ± 0.7
Results of the syntheses where original preparative HLPC method (protocol 1) was used	94	21.1 ± 6.1	99.4 ± 0.2	103 ± 52	2.3 ± 0.7
Results of the syntheses where new preparative HPLC method (protocol 2) was used	56	26.7 ± 5.6	99.3 ± 0.2	150 ± 51	2.8 ± 0.6

A total of 150 successful syntheses divided into subsets of 94 and 56 syntheses. Data are presented as mean ± standard deviation. n = number of trials, RCY = radiochemical yield, RCP = radiochemical purity, A_m_ = molar activity

Table 4 shows a list of all the 12 failed syntheses (12/162) after process validation. List includes type of malfunction, occurrence, cause if identified and a remedy.

**Table 4:** List and analysis of the 12 failed [^11^C]UCB-J syntheses of the 162 reported synthesis

Entry №	Year/№ of [^11^C]UCB-J synthesis	Malfunction of device and/or failed QC	Occurrence description	Cause if identified	Remedy
1	2020/33	**SYN**/**HUC**	Transfer of [^11^C]CH_3_I failed	[^11^C]CH_3_I transfer line displaced	Operator training
2	2021/21	**HC**	Product vial broke in **HC** transfer chute	Defective vial?	None at this time
3	2021/33	**HUC**/**QC**	Product pH out of limit	Valve (V6, Fig. 1B) obstructed	Valve cleaned and inspected
4	2021/34	**HUC**	HPLC injection failed	HPLC inlet frit blocked	Frit change interval shortened
5	2021/36	**HUC**/**QC**	Product pH out of limit	Valve (V6, Fig. 1B) obstructed	Valve cleaned and inspected
6	2021/41	**SYN**	Synthesis of [^11^C]CH_3_I failed	Blockage in Ni-NaOH tube	Tube replaced, service interval shortened
7	2021/52	**SYN**	Synthesis of [^11^C]CH_3_I failed	**SYN** high temperature oven thermoswitch failure	Switch replaced
8	2022/5	**HUC**/**QC**	Product pH out of limit	Valve obstruction identified as septa sliver originating from piercing septa jammed in valve V6 (Fig. 1B). See also entries 3 and 5	Septa needle piercing procedures re-evaluated and changed
9	2022/20	**SYN**	[^11^C]CH_3_I molar activity out of specifications	Gas leakages in **SYN**	Device service procedures re-evaluated, change in synthesis sequence
10	2023/14	**CYCL**/**SYN**	**CYCL** irradiation delayed; timeout for **SYN** liq. N_2_ reservoir	Delay in [^11^C]CO_2_ production	None at this time
11	2023/20	**HUC**	C18 Sep-Pak not installed properly	Operator error in **HUC** preparation prior to synthesis	Operator training
12	2023/33	**HUC**	Line from V2 to reaction vessel not properly installed (Fig. 1A)	Operator error in **HUC** preparation prior to synthesis	Operator training

Acronyms in table are as follows: **SYN**; Synthra MeIplus® [^11^C]CH_3_I synthesis device, **HUC**; In-house built [^11^C]UCB-J synthesis device, **HC**; Hot Cell, **CYCL**; Cyclotron, **QC**; Quality Control

## Discussion

### Initial synthesis optimization

The first improvement to the initial process was to install a system comprising two connected syringes and a small vessel for acid dilution (Fig. 1A) intended for dilution and transfer to the HPLC injection loop. The two 5-mL syringes were connected to one pneumatic piston in a push–pull arrangement. The upper syringe was filled with HPLC eluent (3 mL). Upon piston activation, a small portion of air from the top of syringe pushed the acid (1.6 mL) from the container to the reaction vessel, and then further to the bottom syringe. The acid was followed by the HPLC eluent, which additionally flushed the reaction vessel (Fig. 1A). This system enabled effective liquid transfer from the reaction vessel to the HPLC injection loop, as well as dilution to a larger volume (5 mL) than the reaction vessel (3 mL). The diluted reaction mixture was filtered through a stainless-steel frit, which was located directly before the HPLC loop.

Second improvement to the system was to change the shape and size of the reaction vessel. The 2-mL V-shaped vial was replaced with a 3-mL round-bottomed one. This change ensured better stirring of the reaction mixture during synthesis. In the V-shaped vial, the stirring bar tended to stop spinning when the addition needle was lowered, and would not start again when the needle was removed. Due to the change in the geometry of the reaction vessel, the initial volume of the reaction mixture was increased to maintain the same depth of the liquid for bubbling of the [^11^C]CH_3_I.

Third improvement to the protocol was to change handling of the precursor. As previously reported by Onega et al., the use of commercially available trifluoroborate precursor for [^11^C]UCB-J production gave poor RCYs. Onega et al. developed a method of generating the corresponding boronic acid by treating the precursor with HCl (1 M) prior to synthesis. We tested the use of stronger acid (30% HCl). Our initial results showed substantial improvement when using 30% acid (RCY 37.9 ± 7.5%); therefore, these conditions were chosen for the GMP procedure.

### Synthesis optimization during GMP production

**Fig. 3:**
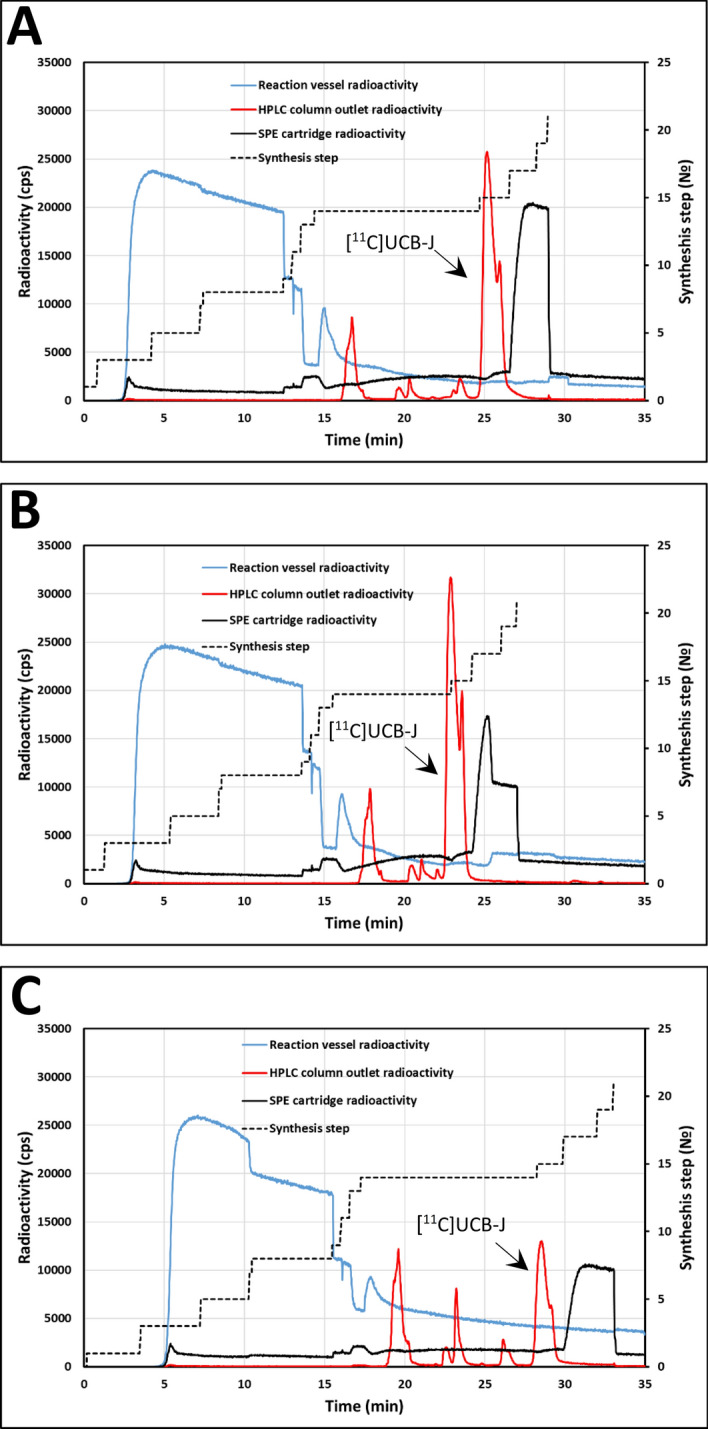
Color-coded data collected from GM tubes (see Fig. 1 for placement) interfaced to the synthesis device. **A** Typical synthesis trends. **B** Trends from synthesis with activity loss from the SPE cartridge. **C** Trends from synthesis with activity loss at the precursor addition step

**Fig. 4:**
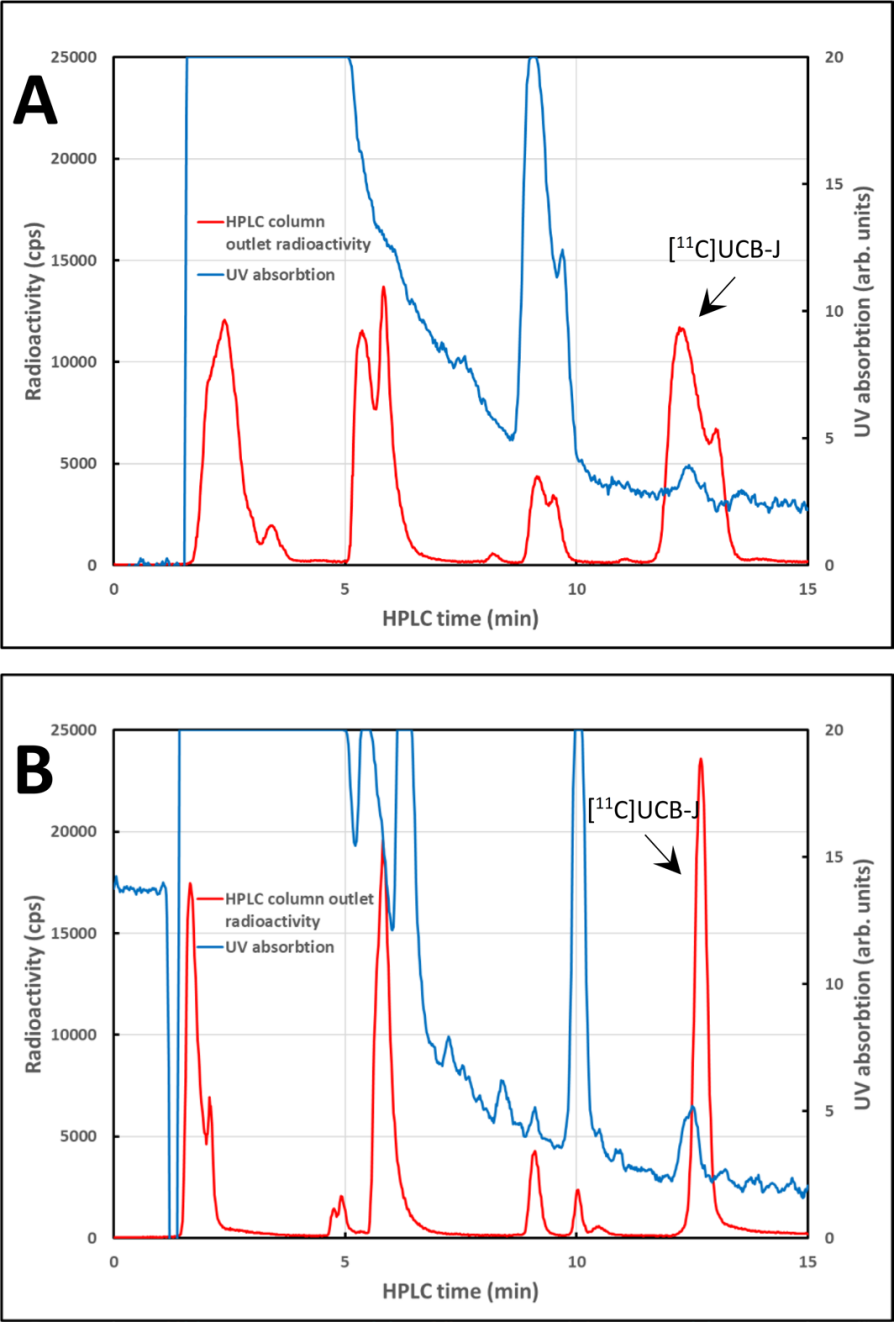
Comparison of the two methods for the radioHPLC purification of the crude [^11^C]UCB-J product. **A** Method previously described by Nabulsi et al. 2016, used in our early developmental studies. **B** Novel separation method developed by us (see section Chromatographic methods)

During clinical production of [^11^C]UCB-J for over four years, several problems were encountered, none of which affect the quality of the final product, but rather the RCY of the synthesis. First, preparative HPLC purification using protocol 1 showed a characteristic shape, with the product peak having a small shoulder peak (Fig. 3A-C, 4A). To ensure high RCP, the second peak was separately collected and QC confirmed it to be the pure product. Thus, a new preparative HPLC method (protocol 2) was developed to better shape the product peak (Fig. 4B). RCY of the batches purified with the new HPLC method (26.7 ± 5.6%) is significantly higher than RCY of the batches purified with the original HPLC method (21.1 ± 6.1%) (Table 3).

Second, it was sometimes found that a portion of product activity which was retained on the SPE cartridge was already eluted when loaded to the cartridge (Fig. 3B). This phenomena was correlated with a very broad HPLC product peak and thus high fraction volume, which resulted in a high concentration of organic solvent during trapping to the SPE cartridge. As this phenomena did not occur in the optimisation syntheses (Table 2), the RCY (Table 2, entry 4) gave higher RCY than the syntheses carried out after process validation (Table 3). To solve this problem, the amount of water used to dilute the collected HPLC fraction was increased from 20 to 35 mL.

Third, an issue occasionally observed was loss of radioactivity during addition of precursor (Fig. 3C). Although the trapping of [^11^C]CH_3_I was effective, we occasionally saw a loss of activity from the reaction vessel during addition of the precursor. Presumably this was caused by incomplete Pd-complex insertion into the carbon-halide bond of [^11^C]CH_3_I. Similar behaviour was also observed by Milicevic Sephton et al. 2020. The loss of activity varied from 0 to 25%. In the majority of syntheses, the activity drop was < 10%, which was considered acceptable. No single reason for such behaviour has been identified. It is likely caused by multiple factors, i.e. the Suzuki cross-coupling reaction is sensitive to air, so the initial reaction mixture must be carefully prepared and properly degassed. Since the reaction mixture is not fully homogenous, constant stirring during the reaction also plays some role. As a remedy, we replaced the initially used cylindrical stirring bar with micro-stirring bar. The cylindrical stirring bar occasionally did not resume spinning once the addition needle was raised. The micro-stirring bar allows constant stirring even when the addition needle is in the solution. Since the start to use the micro-stirring bar, and establishing a reliable procedure for reaction vessel preparation, we have not observed large activity losses, however 27% of the batches have had an activity loss > 10% during addition of the precursor. The average activity loss has been 8.8 ± 2.0%. Moreover, this issue did not affect the quality of the final product. Syntheses with a large activity loss (> 10%), or the first and second issues resulted in significantly (p < 0.05) lower RCY (19.3 ± 5.1%; n = 58) compared to normal syntheses (RCY: 26.7 ± 5.6%, Table 3). The amount of final product was sufficient for clinical applications in all cases.

After the process validation we have had 12 failed syntheses (Table 4). For three of the failed syntheses (Table 4, entries 1, 11 and 12) the cause was identified as operator error and the remedy was operator training. Three of the failures (Table 4, entries 2, 7 and 10) were caused by occurrences beyond our control. The rest of the failures (Table 4, entries 3–6, 8 and 9) were connected to the device preventive maintenance and routine maintenance. Particularly, the occurrences in entries 3, 5 and 8 were identified as caused by piercing of the elution vial septa (Fig. 1B) with a sharp needle, followed by a sliver of the septum entering the vial. Slivers of the septa have caused product pH out of limit three times, due to blockage of the valve V6 (Fig. 1B). We now have a procedure to ensure that these septa slivers are not present in the vial. The occurrences for entries 4, 6 and 9 were followed by changes in routine preventive maintenance. Overall, there has been relatively few failed synthesis (12) comparing to the successful ones (150).

A total of 162 syntheses were performed after process validation. Notably, the described problems affected only the RCY of the synthesis, excluding the 12 failed syntheses, and were not a reason to reject a synthesis batch. As a result of the process re-validation with the new preparative HPLC method there has been significant improvement in the RCYs. In all instances where procedures were changed after process validation or re-validation, documentation was formed according to our change control system.

Overall, the radiosynthesis of [^11^C]UCB-J is not a trivial task. A close observance of GMP regulations and guidelines in all aspects of radiopharmaceutical preparation facilitates this task. Following careful documentation of procedures on all levels in the processes helps to avoid possible pitfalls.

## Conclusions

The presented method enables robust [^11^C]UCB-J production with 26.7 ± 5.6% RCY, corresponding to 2.8 ± 0.6 GBq of product at EOS. The final product fulfils all the specifications set for a clinical tracer. Although [^11^C]UCB-J production is challenging, good and reproducible RCYs can be obtained by following a carefully designed protocol.

## Data Availability

Data are available in the experimental section of the article.

## Abbreviations

A_m_: Molar activity
[^11^C]CO_2_: [^11^C]carbon dioxide
[^11^C]CH_3_I: [^11^C]methyl iodide
CNS: Central nervous system
CP: Chemical purity
[^11^C]UCB-J: (*R*)-1-((3-[^11^C]methylpyridin-4-yl)methyl)-4-(3,4,5-trifluorophenyl)pyrrolidin-2-one
DMF: N,N-dimethylformamide
EOB: End of bombardment
EOS: End of synthesis
EU: European Union
GC: Gas chromatography
GM: Geiger–Müller
GMP: Good manufacturing practice
HPLC: High-performance liquid chromatography
ICP-MS: Inductively coupled plasma-mass spectrometry
LC: Liquid chromatography
PEEK: Polyether ether ketone
PET: Positron emission tomography
Ph. Eur.: European pharmacopoeia
PHT: Pressure hold test
QA: Quality assurance
QC: Quality control
RCY: Radiochemical yield
RCP: Radiochemical purity
RPD: Relative pressure decrease
RPR: Responsible person for radiopharmaceuticals
SD: Standard deviation
SFU: Sterile filtration unit
SOS: Start of synthesis
SPE: Solid phase extraction
SS: Stainless steel
SSRP: Small-scale “in-house” radiopharmaceutical
SV2A: Synaptic vesicle glycoprotein 2A

## References

[CR1] Ametamey SM, Honer M, Schubiger PA. Molecular imaging with PET. Chem Rev. 2008;108:1501–16. 18426240 10.1021/cr0782426

[CR2] Bajjalieh SM, Frantz GD, Weimann JM, McConnell SK, Scheller RH. Differential expression of synaptic vesicle protein 2 (SV2) isoforms. J Neurosci. 1994;14:5223–35. 8083732 10.1523/JNEUROSCI.14-09-05223.1994 PMC6577109

[CR3] Coenen HH, Gee AD, Adam M, Antoni G, Cutler CS, Fujibayashi Y, et al. Consensus nomenclature rules for radiopharmaceutical chemistry—setting the record straight. Nucl Med Biol. 2017;55:v–xi. 29074076 10.1016/j.nucmedbio.2017.09.004

[CR4] Finnema SJ, Nabulsi NB, Eid T, Detyniecki K, Lin S, Chen M-K, et al. Imaging synaptic density in the living human brain. Sci Transl Med. 2016;8: 1–9.348ra96. 10.1126/scitranslmed.aaf6667 27440727

[CR5] Gillings N, Todde S, Behe M, Decristoforo C, Elsinga P, Ferrari V, et al. EANM guideline on the validation of analytical methods for radiopharmaceuticals. EJNMMI Radiopharm Chem. 2020;5:7. 32052212 10.1186/s41181-019-0086-z PMC7016057

[CR6] Gillings N, Hjelstuen O, Ballinger J, Behe M, Decristoforo C, Elsinga P, et al. Guideline on current good radiopharmacy practice (cGRPP) for the small-scale preparation of radiopharmaceuticals. EJNMMI Radiopharm Chem. 2021;6:8. 33580358 10.1186/s41181-021-00123-2 PMC7881071

[CR7] Glantz LA, Lewis DA. Decreased dendritic spine density on prefrontal cortical pyramidal neurons in schizophrenia. Arch Gen Psychiatry. 2000;57:65–73. 10632234 10.1001/archpsyc.57.1.65

[CR8] EU GMP, Volume 4 Good manufacturing practice (GMP) Guidelines. https://ec.europa.eu/health/documents/eudralex/vol-4_en. Accessed 30 April 2020. n.d.

[CR9] Hamos JE, DeGennaro LJ, Drachman DA. Synaptic loss in Alzheimer’s disease and other dementias. Neurology. 1989;39:355–61. 2927643 10.1212/wnl.39.3.355

[CR10] Herth MM, Ametamey S, Antuganov D, Bauman A, Berndt M, Brooks AF, et al. On the consensus nomenclature rules for radiopharmaceutical chemistry – reconsideration of radiochemical conversion. Nucl Med Biol. 2020;93:19–21. 33232876 10.1016/j.nucmedbio.2020.11.003

[CR11] Ishiyama T, Kizaki N, Miyara N, Suzuki A. Synthesis of unsymmetrical biaryl ketones via palladium-catalyzed carbonylative cross-coupling reaction of arylboronic acid with iodoarenes. Tetrahedron Lett. 1993;34:7595–8.

[CR12] Kang HJ, Voleti B, Hajszan T, Rajkowska G, Stockmeier CA, Licznerski P, et al. Decreased expression of synapse related genes and loss of synapses in major depressive disorder. Nat Med. 2012;18:1413–7. 22885997 10.1038/nm.2886 PMC3491115

[CR13] Lynch BA, Lambeng N, Nocka K, Kensel-Hammes P, Bajjalieh SM, Matagne A, et al. The synaptic vesicle protein SV2A is the binding site for the antiepileptic drug levetiracetam. Proc Natl Acad Sci U S A. 2004;101:9861–6. 15210974 10.1073/pnas.0308208101 PMC470764

[CR14] Milicevic Sephton S, Miklovicz T, Russell JJ, Doke A, Li L, Boros I, et al. Automated radiosynthesis of [^11^C]UCB-J for imaging synaptic density by positron emission tomography. J Label Compd Radiopharm. 2020;63:151–8. 10.1002/jlcr.3828 PMC7155065 32027052

[CR15] Miyaura N, Suzuki A. Palladium-catalyzed cross-coupling reactions of organoboron compounds. Chem Rev. 1995;95:2457–83.

[CR16] Nabulsi NB, Hannestad J, Holden D, Mercier J, Najafzadeh S, Lin S, et al. [^11^C]UCB-J: a novel PET tracer for imaging the synaptic vesicle glycoprotein 2A (SV2A). J Nucl Med. 2014;55(Suppl 1):355. 10.2967/jnumed.115.168179 26848175

[CR17] Nabulsi NB, Mercier J, Holden D, Carre S, Najafzadeh S, Vandergeten M-C, et al. Synthesis and preclinical evaluation of 11C-UCB-J as a PET tracer for imaging the synaptic vesicle glycoprotein 2A in the brain. J Nucl Med. 2016;57:777–84. 26848175 10.2967/jnumed.115.168179

[CR18] Onega M, Chong H, Roble A, Plisson C, Huiban M, Mercier J et al. Highly Improved and GMP compliant synthesis of [^11^C]UCB-J: in situ generation of boronic acid precursor. EANM 17;EP-0232

[CR19] Rokka J, Schlein E, Eriksson J. Improved synthesis of SV2A targeting radiotracer [^11^C]UCB-J. EJNMMI Radiopharm Chem. 2019;4:30. 31784919 10.1186/s41181-019-0080-5 PMC6884603

[CR20] Rokka J, Nordeman P, Roslin S, Eriksson J. A comparative study on Suzuki-type 11C-methylation of aromatic organoboranes performed in two reaction media. J Label Compd Radiopharm. 2021;64:447–55. 10.1002/jlcr.3932 34250640

[CR21] Savisto N, Viljanen T, Kokkomäki E, Bergman J, Solin O. Automated production of [^18^F]FTHA according to GMP. J Labelled Comp Radiopharm. 2017;61:84–93. 10.1002/jlcr.3589 29205456

[CR22] Strauss LG, Conti PS. The applications of PET in clinical oncology. J Nucl Med. 1991;32:623–48. 2013803

[CR23] Suzuki A. Synthetic studies via the cross-coupling reaction of organoboron derivatives with organic halides. Pure Appl Chem. 1991;63:419–22.

[CR24] Tang G, Gudsnuk K, Kuo S-H, Cotrina ML, Rosoklija G, Sosunov A, et al. Loss of mTOR-dependent macroautophagy causes autistic-like synaptic pruning deficits. Neuron. 2014;83:1131–43. 25155956 10.1016/j.neuron.2014.07.040 PMC4159743

[CR25] Todde S, Kolenc Peitl P, Elsinga P, Koziorowski J, Ferrari V, Ocak EM, et al. Guidance on validation and qualification of processes and operations involving radiopharmaceuticals. EJNMMI Radiopharm Chem. 2017;2: 8. 29503849 10.1186/s41181-017-0025-9 PMC5824699

[CR26] Van Vliet EA, Aronica E, Redeker S, Boer K, Gorter JA. Decreased expression of synaptic vesicle protein 2A, the binding site for levetiracetam, during epileptogenesis and chronic epilepsy. Epilepsia. 2009;50:422–33. 18717715 10.1111/j.1528-1167.2008.01727.x

